# Supplementary material for: Doxycycline has distinct apicoplast-specific mechanisms of antimalarial activity
Source: eLife. 2020 Nov 2;9:e60246. doi: 10.7554/eLife.60246 (PMC7669263; doi:10.7554/eLife.60246)
Supplement: Transparent reporting form [file elife-60246-transrepform.pdf]

eLIFE
1st Floor
24 Hills Road
Cambridge CB2 1JP, UK
P 01223 855340
W elifesciences.org
T @elife

eLife's transparent reporting form

We encourage authors to provide detailed information *within their submission* to facilitate the interpretation and replication of experiments. Authors can upload supporting documentation to indicate the use of appropriate reporting guidelines for health-related research (see EQUATOR Network), life science research (see the BioSharing Information Resource), or the ARRIVE guidelines for reporting work involving animal research. Where applicable, authors should refer to any relevant reporting standards documents in this form.

If you have any questions, please consult our Journal Policies and/or contact us: editorial@elifesciences.org.

Sample-size estimation

- You should state whether an appropriate sample size was computed when the study was being designed
- You should state the statistical method of sample size computation and any required assumptions
- If no explicit power analysis was used, you should describe how you decided what sample (replicate) size (number) to use

Please outline where this information can be found within the submission (e.g., sections or figure legends), or explain why this information doesn't apply to your submission:

Sample replicate numbers were determined based on prior experience with the experiments that were performed and on practical considerations for carrying out these experiments. N numbers and statistical tests are described in the manuscript text, methods section, and figure legends.

Replicates

- You should report how often each experiment was performed
- You should include a definition of biological versus technical replication
- The data obtained should be provided and sufficient information should be provided to indicate the number of independent biological and/or technical replicates
- If you encountered any outliers, you should describe how these were handled
- Criteria for exclusion/inclusion of data should be clearly stated
- High-throughput sequence data should be uploaded before submission, with a private link for reviewers provided (these are available from both GEO and ArrayExpress)

Please outline where this information can be found within the submission (e.g., sections or figure legends), or explain why this information doesn't apply to your submission:

eLife Sciences Publications, Ltd is a limited liability non-profit non-stock corporation incorporated in the State of Delaware, USA, with company number 5030732, and is registered in the UK with company number FC030576 and branch number BR015634 at the address 1st Floor, 24 Hills Road, Cambridge CB2 1JP | August 2014

We defined biological and technical replicates following the conventions of Blainey et al. (2014) *Nature Methods*, 11: 879-880. Biological replicates consisted of distinct parasite sample wells set-up and monitored in parallel for the duration of a given growth assay. Numbers of replicates for each experiment are given in the Methods section and in figure legends.

1. Growth assays: Individual data points in plots are the average ± standard deviation of 2-4 biological replicates monitored in parallel. Each growth experiment was independently performed 2-5 times on different days with different blood batches. No outlying data points have been excluded in plots.

2. Microscopy analysis: Microscopy analyses were conducted in 2-3 repeat experiments on different days with different blood batches. For quantification, phenotypes were binned in discrete categories and counted for 20-40 total parasites across these independent experiments.

Statistical reporting

- Statistical analysis methods should be described and justified
- Raw data should be presented in figures whenever informative to do so (typically when N per group is less than 10)
- For each experiment, you should identify the statistical tests used, exact values of N, definitions of center, methods of multiple test correction, and dispersion and precision measures (e.g., mean, median, SD, SEM, confidence intervals; and, for the major substantive results, a measure of effect size (e.g., Pearson's r, Cohen's d)
- Report exact p-values wherever possible alongside the summary statistics and 95% confidence intervals. These should be reported for all key questions and not only when the p-value is less than 0.05.

Please outline where this information can be found within the submission (e.g., sections or figure legends), or explain why this information doesn't apply to your submission:

Descriptions of statistical tests are given in Methods and in figure legends. For experiments involving repeat measurements, an average ±standard deviation is reported. Differences in averages from repeat measurements were analyzed for significance by two-tailed unpaired t-test. P values for all such tests are explicitly stated in figures.

(For large datasets, or papers with a very large number of statistical tests, you may upload a single table file with tests, Ns, etc., with reference to sections in the manuscript.)

Group allocation

- Indicate how samples were allocated into experimental groups (in the case of clinical studies, please specify allocation to treatment method); if randomization was used, please also state if restricted randomization was applied
- Indicate if masking was used during group allocation, data collection and/or data analysis

Please outline where this information can be found within the submission (e.g., sections or figure legends), or explain why this information doesn't apply to your submission:

For microscopy analyses, apicoplast morphologies based on ACP_L-GFP signal were scored as elongated, punctate, or dispersed as indicated in the examples shown.

Additional data files ("source data")

- We encourage you to upload relevant additional data files, such as numerical data that are represented as a graph in a figure, or as a summary table
- Where provided, these should be in the most useful format, and they can be uploaded as "Source data" files linked to a main figure or table
- Include model definition files including the full list of parameters used
- Include code used for data analysis (e.g., R, MatLab)
- Avoid stating that data files are "available upon request"

Please indicate the figures or tables for which source data files have been provided:

All data is shown in figures and supplemental figures in our manuscript. Source data for microscopy analyses in figure supplements is provided in an Excel file.

eLife Sciences Publications, Ltd is a limited liability non-profit non-stock corporation incorporated in the State of Delaware, USA, with company number 5030732, and is registered in the UK with company number FC030576 and branch number BR015634 at the address 1st Floor, 24 Hills Road, Cambridge CB2 1JP | August 2014
